# Influence of specific HSP70 domains on fibril formation of the yeast prion protein Ure2

**DOI:** 10.1098/rstb.2011.0410

**Published:** 2013-05-05

**Authors:** Li-Qiong Xu, Si Wu, Alexander K. Buell, Samuel I. A. Cohen, Li-Jun Chen, Wan-Hui Hu, Sarah A. Cusack, Laura S. Itzhaki, Hong Zhang, Tuomas P. J. Knowles, Christopher M. Dobson, Mark E. Welland, Gary W. Jones, Sarah Perrett

**Affiliations:** 1 National Laboratory of Biomacromolecules, Institute of Biophysics, Chinese Academy of Sciences, 15 Datun Road, Chaoyang, Beijing 100101, People's Republic of China; 2 Nanoscience Centre, University of Cambridge, 11 JJ Thomson Avenue, Cambridge CB3 0FF, UK; 3 Department of Chemistry, University of Cambridge, Lensfield Road, Cambridge CB2 1EW, UK; 4 Department of Biology, National University of Ireland Maynooth, Maynooth, County Kildare, Republic of Ireland; 5 Graduate University of the Chinese Academy of Sciences, 19 Yuquan Road, Shijingshan, Beijing 100049, People's Republic of China

**Keywords:** prion, amyloid, Ure2p, Ssa1p, chaperone, quartz crystal microbalance

## Abstract

Ure2p is the protein determinant of the *Saccharomyces cerevisiae* prion state [*URE3*]. Constitutive overexpression of the HSP70 family member *SSA1* cures cells of [*URE3*]. Here, we show that Ssa1p increases the lag time of Ure2p fibril formation *in vitro* in the presence or absence of nucleotide. The presence of the HSP40 co-chaperone Ydj1p has an additive effect on the inhibition of Ure2p fibril formation, whereas the Ydj1p H34Q mutant shows reduced inhibition alone and in combination with Ssa1p. In order to investigate the structural basis of these effects, we constructed and tested an Ssa1p mutant lacking the ATPase domain, as well as a series of C-terminal truncation mutants. The results indicate that Ssa1p can bind to Ure2p and delay fibril formation even in the absence of the ATPase domain, but interaction of Ure2p with the substrate-binding domain is strongly influenced by the C-terminal lid region. Dynamic light scattering, quartz crystal microbalance assays, pull-down assays and kinetic analysis indicate that Ssa1p interacts with both native Ure2p and fibril seeds, and reduces the rate of Ure2p fibril elongation in a concentration-dependent manner. These results provide new insights into the structural and mechanistic basis for inhibition of Ure2p fibril formation by Ssa1p and Ydj1p.

## 1. Introduction

Prions are infectious proteins, accounting for a group of invariably fatal neurodegenerative diseases in mammals, including Creutzfeldt–Jakob disease in humans, and bovine spongiform encephalopathy and scrapie in animals [1]. These prion diseases are closely associated with a host-derived protein called PrP, whose modified isoform is generally thought to be the principal constituent of the prion particle [1,2]. Normal PrP converts to infectious pathogenic PrP^Sc^ through a refolding process in which the α-helix and random coils rearrange into a β-sheet structure [3,4]. The discovery that the *Saccharomyces cerevisiae* genetic element [*URE3*] consists of an altered form of the protein that has the ability to convert normal Ure2p into the altered form [5], broadened the concept of prions to include transmission of non-fatal or even favourable traits. The expansion of the prion concept to fungal proteins has allowed significant progress in mechanistic understanding of the prion phenomenon, with yeast genetics providing a powerful complement to biophysical approaches [6].

Ure2p is a 354-residue protein, composed of two structural domains with distinct functions [7]. The N-terminal domain is relatively flexible and protease-sensitive [8,9], and conveys the ability to switch to a prion state *in vivo* and to form amyloid-like fibrils *in vitro* [10–13]. Recent results have defined a short fibril-forming peptide region within the N-terminal domain as a potential initiation point for amyloid formation [14]. The crystal structure of the C-terminal domain shows similarity to glutathione *S*-transferases (GSTs) [15,16], although the protein lacks typical GST activity [17,18]. However, Ure2p shows both glutathione-dependent peroxidase activity [19] and thiol-disulfide oxidoreductase activity [20]. Further, typical GST activity can be restored by single-site mutation at the enzyme active site [21]. The ability of Ure2p to bind to Gln3p, and hence exert its nitrogen catabolite repression activity, is lost when soluble Ure2p converts into the aggregated [*URE*3] prion form [10], but the anti-oxidant activity of Ure2p is maintained in prion strains *in vivo* [18] and in amyloid-like fibrils *in vitro* [19–21].

Heat-shock proteins play an important role in preventing protein misfolding and aggregation, and the cytosolic HSP70 family is one of the major classes of chaperones involved in regulating prion propagation [22]. The Ssa subfamily of HSP70 chaperones comprises four members (Ssa1–4) and is essential for cell viability [23]. Ssa proteins are involved in a variety of cellular processes, such as translation, translocation and general protein folding [24–26]. *SSA1* and *SSA2* are constitutively expressed and their gene products are 96 per cent identical, whereas *SSA3* and *SSA4* are heat-shock-inducible and their gene products are both 80 per cent identical to Ssa1p and Ssa2p.

**Figure 1.**
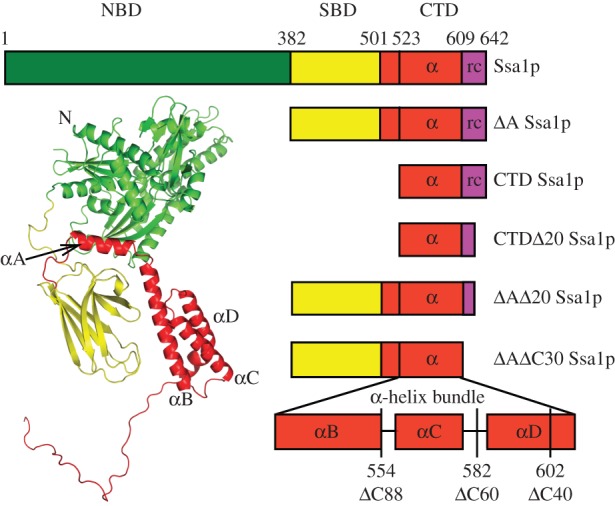
Schematic of Ssa1p domain structure and deletion mutants used in this study. The three-dimensional predicted full-length Ssa1p structure was modelled using RossettaDock protein–protein docking software (www.rosettacommons.org) based on the structures of bovine Hsc70 (1yuw) and DnaK (1dkx).

The only Ssa protein structures published to date are that of a short C-terminal peptide of Ssa1p in complex with Sis1p, a co-chaperone of HSP70; or with Tom71, a component of the mitochondrial translocon [27–29]. However, the HSP70 family shares a highly conserved architecture, consisting of an N-terminal nucleotide-binding domain (NBD), a substrate-binding domain (SBD) and a C-terminal lid domain (CTD) [30–34]. Owing to its ability to hydrolyse adenosine triphosphate (ATP) into adenosine diphosphate (ADP), the NBD is also termed the ATPase domain. The SBD is a β-sandwich structure containing a long peptide binding groove, whereas the α-helical bundle C-terminal domain acts as a lid, stabilizing the complex of HSP70 and substrate [35]. A model of the Ssa1p structure and the sequence positions of its domain boundaries are illustrated in figure 1.

The role of the ATPase activity of HSP70 is to modulate its substrate binding affinity and to facilitate substrate release. In the ATP binding state, HSP70 tends to release the substrate, whereas in the ADP binding state, its affinity for the substrate is relatively high [36–38]. The intrinsic ATPase activity of Ssa1p is stimulated by co-chaperones, including Ydj1p [39–42]. ATP binding induces conformational changes in the adjacent domains, including movement of the lid so that the substrate binding groove becomes uncovered. By contrast, substrate binding causes ATP hydrolysis and closure of the lid over the substrate binding groove [43,44]. Thus, the ATPase cycle contributes to the efficiency of the essential cellular functions of HSP70 by promoting substrate binding and release. However, current understanding of the ATPase cycle of HSP70 has primarily been derived from studies using small peptide substrates, whereas recent results suggest that the structural changes associated with substrate binding may be different for larger substrates [45]. Interestingly, HSP70 is able to bind and prevent aggregation of substrates such as alpha-synuclein even in the absence of any nucleotide [46–48], suggesting that the intrinsic chaperone activity of HSP70 may still be maintained even without energy generated from ATP hydrolysis. Recent mutagenesis studies on DnaK show that the ability to refold luciferase correlates poorly with the rate of ATP turnover [49]. Further, certain mutants of DnaK can refold luciferase normally in the absence of any significant ATP turnover, and also complement a *ΔdnaK* strain of *Escherichia coli*. This indicates that removal of the ATPase function of HSP70 does not necessarily ablate its ability to protect proteins from misfolding and aggregation *in vitro* and *in vivo* [49].

Ssa1p shows differing effects on the yeast prions [*PSI*^+^] and [*URE3*] *in vivo*. Constitutive overexpression of Ssa1p but not the nearly identical Ssa2p cures cells of [*URE3*] [50]. Additionally, several mutants of Ssa1p and Ssa2p have been identified that are capable of impairing [*URE3*] propagation [51]. With regard to [*PSI*^+^], excess Ssa1p protects [*PSI*^+^] from curing induced by overexpression of HSP104 [52]. [*PSI*^+^] stability is normal in strains that lack *SSA1* or *SSA2* [53] and overexpression of Ssa1p increases de novo [*PSI*^+^] formation [54]. However, recent evidence suggests that overexpression of either Ssa1p or Ssa2p can cure some variants of [*PSI*^+^] by increasing the size of prion aggregates and hence hindering prion transmission to daughter cells [55]. *In vitro* studies show that Ssa1p alone does not affect Sup35p fibril assembly, but can block fibril formation in the presence of either of its HSP40 co-chaperones, Ydj1p or Sis1p [56]. The action of HSP104p in promoting fibril formation of Sup35p is reversed in the presence of complexes of Ssa1p with its co-chaperones [56]. It has recently been reported that Ssa1p inhibits Ure2p amyloid fibril formation in the presence of ATP [57,58]. The co-chaperone Ydj1p can also inhibit Ure2p fibril formation itself [57,59]. The previous studies indicate that inhibitory effects of both Ssa1p and Ydj1p on Ure2p fibril formation *in vitro* depend on the interaction of the chaperone with the C-terminal region of Ure2p [57,59,60].

The mechanism of how Ssa1p inhibits Ure2p fibril formation is complex and is not yet fully understood. It remains unclear how specific domains of Ssa1p function independently or in cohort to produce the observed inhibition of fibril formation of Ure2p. To address these questions, we used the power of *in vitro* methods to dissect out the relative contribution of the different Ssa1p structural domains, by investigating the effect of a series of deletion mutants of Ssa1p on Ure2p fibril formation. We also investigated in further detail the mode and mechanism of interaction between Ssa1p and Ure2p.

## 2. Material and methods

### (a) Materials

1,4-Dithiothreitol (DTT), β-mercaptoethanol, Tris, thioflavin T (ThT), ATP, ADP, creatine kinase (CK) and creatine phosphate (CP) were purchased from Sigma. TEV (tobacco etch virus) protease was from Invitrogen. Recombinant firefly luciferase and luciferase assay kit were from Promega. All other reagents were local products of analytical grade. Deionized distilled water was used throughout.

### (b) Protein expression and purification

Ure2p was expressed and purified under native conditions as described previously [8], except that a French press was used to disrupt the cells. Ydj1p was purified as previously described and the His-tag was left intact [59]. The Ssa1p expression plasmid (pPROEX-HTb-Ssa1) was provided by Prof. Susan Lindquist (available from Adgene as plasmid 1231). The Ssa1p domain-deletion mutants were derived from the Ssa1p gene. The boundaries were determined based on tertiary structure predictions, using the partial structures of bovine Hsc70 [30] and DnaK [35] (figure 1). The Ssa1p protein and its truncation mutants were produced and purified as described [61,62], with some modifications [63]. In brief, the harvested cells were lysed using a JNBIO JN-3000 PLUS high-pressure cell press in buffer A (40 mM Hepes–KOH buffer, pH 7.4, containing 300 mM KCl, 5% glycerol and 2 mM β-mercaptoethanol) containing 20 mM imidazole, and the debris was removed by centrifugation (35 000*g*, 30 min). The supernatant was then loaded onto a Ni affinity column (chelating sepharose fast-flow resin; Pharmacia/GE) and washed with buffer A containing 100 mM imidazole. Protein was eluted using buffer A containing 350 mM imidazole and dialysed into 50 mM Tris–HCl buffer pH 8.4 containing 150 mM KCl, 5 mM MgCl_2_ and 1 mM DTT with or without 5 per cent glycerol (henceforth abbreviated to ‘Tris buffer pH 8.4’). Treatment with TEV protease to remove the His-tag was followed by a further round of purification by Ni affinity chromatography. It was found that the presence or absence of the His-tag had no effect on the ability of Ssa1p to inhibit Ure2p fibril formation. Therefore, the His-tagged protein was used in subsequent experiments. *In vivo*, when it is the sole HSP70-Ssap in the cell, His-tagged Ssa1p is well expressed and is able to provide essential cellular functions [64]. In order to confirm that our purified Ssa1p had normal ATPase and chaperone functions, we measured its intrinsic ATPase activity in the presence and absence of co-chaperone Ydj1 [65,66] (see the electronic supplementary material, figure S1*a*) and its ability to refold luciferase [67,68] (see the electronic supplementary material, figure S1*b*).

All protein concentrations are given in terms of monomer and were determined by the absorbance at 280 nm using calculated extinction coefficients of 48 220 M^−1^ cm^−1^ for Ure2p [8], 19 770 M^−1^ cm^−1^ for Ydj1p [59], 23 970 M^−1^ cm^−1^ for Ssa1p and 14 650 M^−1^ cm^−1^ for *Δ*A Ssa1p. The concentration of Ssa1p and its mutants was also confirmed by bicinchoninic acid assay kit (Pierce).

### (c) *In vitro* amyloid fibril formation

The formation of Ure2p fibrils was monitored by ThT binding fluorescence as previously described [13,69] on a Hitachi FL-4500 fluorimeter. Ure2p amyloid fibrils were prepared by incubation of Ure2p at 30°C in either a Sim International SI-300 incubator shaking at 220 rpm or in an Innova 4230 incubator shaking at 300 rpm, and ThT fluorescence was monitored discontinuously as described [13,69]. Alternatively, samples were incubated in 96-well plates in a Perkin-Elmer EnSpire multimode plate reader shaking at 900 rpm and 1 mm amplitude at 30°C, with recording continuously at 5–15 min intervals by including 10 μM ThT in the protein sample. The trends reported were reproducible by both these methods of incubation and monitoring. A range of incubation conditions were tested including: ‘Tris buffer pH 8.4’ (see above), ‘Tris buffer pH 7.5’ (50 mM Tris–HCl buffer, pH 7.5, containing 150 mM KCl, 5 mM MgCl_2_ and 1 mM DTT) and ‘phosphate buffer pH 8.4’ (50 mM phosphate buffer, pH 8.4, containing 150 mM KCl, 5% glycerol and 1 mM DTT) at 30°C. The stability of Ure2p is higher in Tris buffer than in phosphate buffer, and higher at pH 8.4 than at pH 7.5, with the result that the stability of Ure2p is similar in the latter two buffers [69]. In general, a stronger inhibition effect by the chaperones was observed in phosphate buffer and so this buffer was preferred. However, phosphate buffer was avoided for experiments performed in the presence of nucleotides. The trends reported were reproducible under the following range of conditions: Tris or phosphate buffer, with or without 5 per cent glycerol, pH 7.5–8.4. Representative data are displayed and precise experimental details are given in the figure legends. As the absolute lag time of Ure2p fibril formation is sensitive to even small changes in experimental conditions, such as temperature and shaking speed, experiments were performed in parallel wherever possible. Data shown in a single figure panel were always derived from a single parallel experiment using a single batch of Ure2p or chaperone, and each data point represents the average of at least three replicate samples. Error bars indicate the standard error of the mean. Where indicated, the curves were normalized to the same final fluorescence plateau value to facilitate comparison and/or data fitting. Unless otherwise indicated, a fit of the data to a Boltzman sigmoidal curve is shown. An ATP-regenerating system (50 μg ml^−1^ CK and 8 mM CP) was included for the experiments involving ATP.

### (d) Kinetic analysis of fibril growth curves

**Figure 2.**
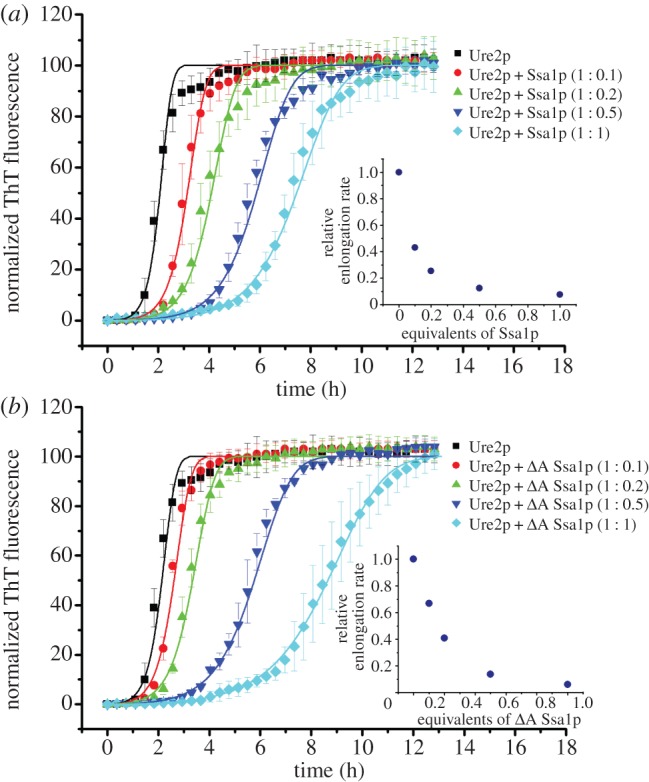
Ssa1p inhibits fibril formation of Ure2p in a concentration-dependent manner. Incubation was in a Perkin-Elmer EnSpire multimode plate reader at 30°C in Tris buffer pH 7.5. Fibril formation was monitored by *in situ* measurement of ThT fluorescence. The error bars represent the standard error of the mean. Data were normalized. Other details are as described in §2. Ure2p (30 μM) in the absence or presence of different molar ratios of (*a*) WT Ssa1p or (*b*) *Δ*A Ssa1p, as indicated. The data are fitted (solid lines) globally with an analytical model of breakable fibril assembly (see §2); this constrained fit, where only the rate of elongation is affected by the chaperone, is able to describe the entire dataset. The insets show the variation in the relative elongation rate with chaperone concentration, as determined by the fitted rate parameters.

The fibril formation data (figure 2) were fitted to an analytical solution of the kinetics of breakable filament assembly [70–74]. Within this framework, the evolution of the fibril mass concentration is given in terms of the rate constants for primary nucleation (*k*_n_), fibril elongation (*k*_+_) and fibril fragmentation (*k*_−_), as well as the concentration (*M*_0_) and average length (*L*_0_) of pre-formed seed material if present at zero time. In the case of a reaction with no pre-formed seeds, the analytical solution given in references [70–72] predicts that the measured fluorescent signal depends upon only two combinations of these parameters, *k*_n_/*k_−_* and *k*_+_*k*_−_*,* rather than each of them individually, reducing the freedom of fits to the model. In figure 2*a*, the data are fitted globally to the solution from [70–72] such that the parameter *k*_n_/*k*_−_ is fixed globally to a single value across all of the chaperone concentrations (including for the data in the absence of chaperone), whereas the second parameter *k*_+_*k*_−_ is allowed to vary for the data at each individual chaperone concentration. An equivalent analysis to that in figure 2*a* is performed in figure 2*b*, with *k*_n_/*k*_−_ fixed globally across all chaperone concentrations to the best global value and *k*_+_*k*_−_ allowed to vary for each of the chaperone concentrations. These constrained fits, where *k*_n_/*k*_−_ is fixed to a single value over all chaperone concentrations, effectively allow only the elongation rate to vary between different concentrations of chaperone (assuming *k*_−_ and *k*_n_ are not correlated), revealing that the effect of Ssalp can be captured primarily by a change in the rate with which fibrils elongate, with any variations in the rates of the other processes being less important in defining the observed changes in growth profiles. The insets in figure 2*a* and *b* show respectively the variation with Ssa1p and *Δ*A Ssa1p concentration of the relative elongation rates as determined from the fitted rate parameters.

### (e) Electron microscopy

A 10 μl drop of the amyloid fibril formation sample was placed onto a copper grid coated with a Formvar film. The sample was then negatively stained with uranyl acetate (2% w/v) and observed using a Philips Tecnai 20 electron microscope.

### (f) Far-UV circular dichroism spectroscopy

CD spectra were acquired on a Pistar-180 spectrometer (Applied Photophysics Ltd, UK). Spectra were measured for each protein sample in Tris buffer pH 8.4 within the range from 195 to 260 nm in a 0.1 cm cuvette at 25°C. In each case, three to five spectra were acquired and then averaged.

### (g) Seed preparation

Ure2p (50 μM) was incubated at 4°C in Tris buffer pH 8.4 without shaking until fibril formation was complete (approx. 7 days). The mature fibrils were then sonicated using a probe sonicator (Sonics and Materials VCX750) for a total of 8 s (in 1 s bursts) at 23 per cent sonication power to obtain seeds.

### (h) Quartz crystal microbalance measurements

The attachment of the Ure2p fibril seeds to the gold surface of the quartz crystal microbalance (QCM) sensor was carried out in a similar manner to the protocol described previously [75]. A solution of 5 μM Ure2p sonicated fibril seeds was mixed with a 10-fold molar excess of Traut's reagent (2-iminothiolane–HCl) and incubated for 5 min. Then, 100 μl of the mixture was added onto the gold surface of a QCM sensor for 1.5 h. The surface was rinsed with ddH_2_O. Finally, the remaining gold surface was passivated via incubation for 1 h with a 0.1 per cent solution of methoxy polyethylene glycol thiol (Polypure, Oslo, Norway). The sensor was rinsed again with water and inserted into the microbalance flow cell (Q-sense, E4, Q-Sense; Västra Frölunda, Sweden) and was ready to use after equilibration with Tris buffer pH 8.4 overnight. For measurements, 300 μl of 1 μM Ure2p solution was introduced into the tubing and flow cell with a peristaltic pump at a flow rate of 150 μl min^−1^. The pump was then switched off, and the measurement was performed in the absence of flow. In order to evaluate the seed fibril elongation rate under a given set of conditions, the steady-state part of the induced frequency shift was fitted to a linear function, and the slope was taken to be proportional to the elongation rate [76].

### (i) Atomic force microscopy

Atomic force microscopy (AFM) was used to assess the morphology of the seeds prepared for attachment to the QCM sensor and to observe the final state of the QCM-sensor surface after measurements were complete. The Ure2p fibril seed sample (10 μl) was dropped onto a freshly cleaved mica surface and allowed to stand for 5 min. The mica was then washed with double-distilled water to remove salts and dried with nitrogen gas. The QCM sensor was also washed with double-distilled water before use and dried with nitrogen gas. A PicoPlus AFM (Molecular Imaging, Tempe, AZ, USA) was used for imaging.

### (j) Dynamic light scattering

The dynamic light scattering (DLS) signal of 10 μM Ure2p and 10 μM *Δ*A Ssa1p, individually or as a mixture, was monitored using a Malvern Zetasizer Nano ZS instrument (Malvern, UK) at room temperature in Tris buffer pH 8.4.

### (k) Pull-down assay

A 100 μl mixture of 10 μM Ure2p seeds and 20 μM wild-type (WT) or *Δ*A Ssa1p was incubated in Tris buffer pH 8.4 in a 30°C water bath for 2 h and then loaded onto the surface of 0.9 ml of a 40 per cent sucrose solution prior to centrifugation at 61 000 rpm for 30 min in a S140AT rotor at 4°C using a Hitachi CS150GXL centrifuge. The pellets were washed twice and resuspended in Tris buffer pH 8.4 containing 1 per cent sodium dodecyl sulfate (SDS), and boiled for 10 min before applying to the SDS-polyacrylamide gel electrophoresis (PAGE).

## 3. Results

### (a) Ssa1p inhibits Ure2p amyloid fibril formation

ThT fluorescence is widely used to probe the kinetics of Ure2p amyloid fibril formation, which typically shows a sigmoidal curve, with an initial lag phase, a subsequent exponential phase, and finally a plateau phase [69,77,78]. The ThT fluorescence assay was used to monitor the fibril formation of Ure2p in the presence or absence of Ssa1p and its mutants.

**Figure 3.**
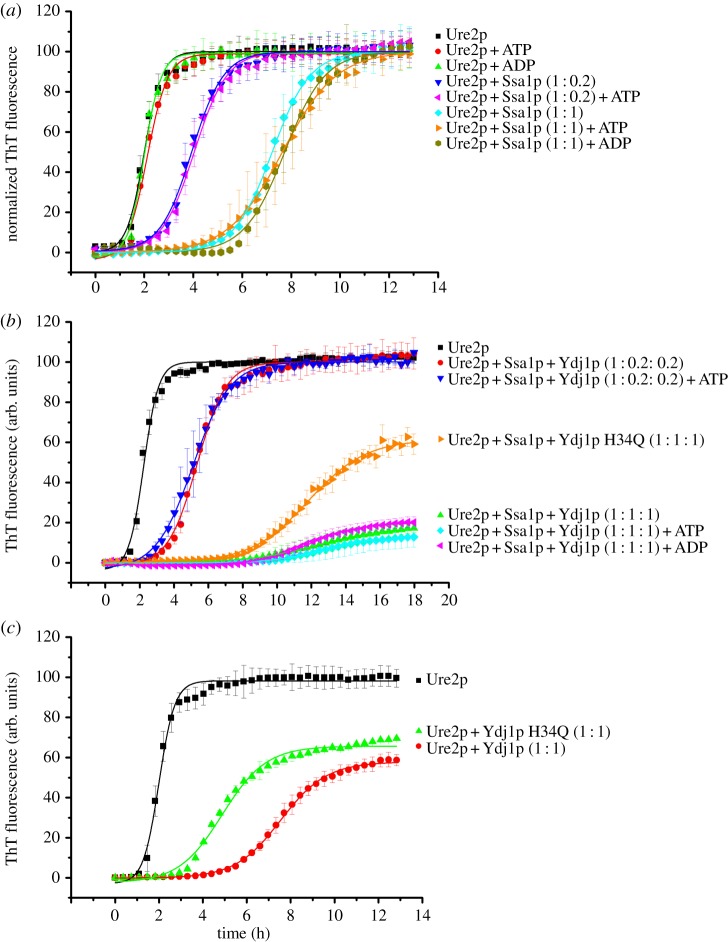
The inhibition of Ure2p amyloid fibril formation by Ssa1p is nucleotide-independent in the presence and absence of Ydj1p. Experimental conditions were the same as described in the legend to figure 2. The error bars represent the s.e. of the mean. A sigmoidal fit of the data is shown. (*a*) Ure2p (30 μM) in the absence or presence of different molar ratios of Ssa1p, with 1 mM ATP or ADP or without nucleotide, as indicated. (*b*) Ure2p (30 μM) in the absence or presence of different molar ratios of Ssa1p and WT or H34Q Ydj1p, with or without 1 mM ATP or ADP, as indicated. Data are shown without normalization. (*c*) Ure2p (30 μM) in the absence or presence of an equimolar ratio of WT or H34Q Ydj1p, as indicated.

Different concentrations of Ssa1p were added to the ThT assay, such that the molar ratio of Ssa1p/Ure2p ranged from 0.1 to 1 molar equivalents. Addition of Ssa1p caused the lag time to be prolonged in a concentration-dependent manner (figure 2*a*). The degree of inhibition of Ure2p fibril formation by Ssa1p was found to be similar in the presence or absence of ATP or ADP (figure 3*a*), indicating that inhibition does not require the presence of nucleotide. Even when lower concentrations of Ssa1p (0.2 molar equivalent of Ssa1p to Ure2p) were used, no significant effect of the presence or absence of ATP was observed (figure 3*a*). The morphology of fibrils harvested during the plateau phase was indistinguishable in the presence and absence of chaperone, and with or without ATP or ADP, as judged from electron microscopy (data not shown), suggesting that Ssa1p simply delays the process of fibril formation without arresting it completely.

The addition of pre-formed seed fibrils of Ure2p shortens or circumvents the lag phase; however, when Ssa1p was added alongside seed fibrils, the presence of Ssa1p had a similar inhibitory effect on the fibril formation kinetics as in unseeded solution (not shown). As further controls, incubation of bovine serum albumin (BSA) with Ure2p had no effect on Ure2p fibril formation and incubation of Ssa1p alone showed no ThT fluorescence increase under the same conditions (not shown).

To investigate at which stages of Ure2p fibril formation Ssa1p acts, we delayed the addition of Ssa1p to varying degrees (see the electronic supplementary material, figure S2). Ssa1p was added at the start of the experiment or at later time points, corresponding to the beginning, mid-lag time, early-exponential and late-exponential phases of fibril formation; for the control sample, an equivalent volume of buffer was added instead. The lag time was delayed to a similar extent when Ssa1p was added at the beginning or the middle of the lag phase (see the electronic supplementary material, figure S2*a*,*b*), and a significant delay was still observed when Ssa1p was added at the beginning of the exponential phase (see the electronic supplementary material, figure S2*c*), suggesting that Ssa1p interacts with species of Ure2p that are significantly populated during and beyond the lag phase, such as native protein and/or fibril seeds. Our results also indicate that Ssa1 does not disaggregate mature fibrils of Ure2p, even in the presence of ATP, consistent with previous results [57].

As the ATPase activity of Ssa1p is stimulated by the co-chaperone Ydj1p [39,42] (see the electronic supplementary material, figure S1*a*), we also examined the effect of nucleotide on inhibition of Ure2p fibril formation by Ssa1p in the presence and absence of Ydj1p. The results show that even in the presence of Ydj1p, the presence or absence of nucleotide has no detectible effect on the ability of Ssa1p to inhibit the fibril formation of Ure2p (figure 3*b*). The lag time of Ure2p fibril formation in the presence of Ssa1p and Ydj1p (figure 3*b*) is longer than that in the presence of Ssa1p alone (figure 3*a*), which is attributed to the inhibition effect of Ydj1p, because Ydj1p itself can lengthen the lag time of Ure2p fibril formation (figure 3*c*), as reported previously [57,59]. The J domain of Ydj1p, which can stimulate the ATPase activity of Ssa1p, is important for curing of [*URE3*] *in vivo*; hence, it has been suggested that the curing effect of Ydj1p on [*URE3*] is indirect and occurs via its effect on Ssa1p [79,80]. We therefore tested the Ydj1p mutant H34Q, which is deficient in stimulating the ATPase activity of HSP70 [81,82] and also disables curing of [*URE3*] by Ydj1p [79,80]. We found that Ydj1p H34Q shows significantly weaker inhibition of Ure2p fibrillation *in vitro* compared with WT Ydj1p, both in the presence (figure 3*b*) and absence (figure 3*c*) of Ssa1p, which could account for the weaker curing effect of Ydj1p H34Q *in vivo* [79,80].

### (b) The role of the ATPase and C-terminal lid domains of Ssa1p in the inhibition of Ure2p fibril formation

The lack of effect of the presence of nucleotides in the inhibition of Ure2p fibril formation by Ssa1p suggests that this effect may arise predominantly from the interaction between Ure2p and the C-terminal region of Ssa1p. In order to investigate the structural basis of the inhibition effect, we designed a series of deletion or truncation mutants of Ssa1p. Given the high similarity of the primary structure of HSP70 proteins, we used the bovine Hsc70 (NBD, SBD and part of CTD) and DnaK (SBD and CTD) to model the structure of Ssa1p (figure 1). Based on the predicted structure, we designed the truncation mutant *Δ*A Ssa1p, which consists of residues 382–642 and lacks the ATPase domain (figure 1 and table 1). When Ure2p fibril formation was carried out in the presence of *Δ*A Ssa1p, the results were similar to those for WT Ssa1p (figure 2), consistent with the lack of nucleotide dependence of the effect of WT Ssa1p (figure 3).

**Table 1.** Summary of Ssa1p deletion mutants used in this study. NBD, nucleotide binding domain; SBD, substrate binding domain; CTD, C-terminal domain; *α*, *α*-helix; rc, random coil. See also figure 1.

protein	residues	domains
WT Ssa1p	1–642	NBD, SBD, CTD
*Δ*A Ssa1p	382–642	SBD, CTD
*Δ*A*Δ*C20 Ssa1p	382–622	SBD, CTD (*α*A-D, part of rc)
*Δ*A*Δ*C30 Ssa1p	382–612	SBD, CTD (*α*A-D)
*Δ*A*Δ*C40 Ssa1p	382–602	SBD, CTD (*α*A-C, part of *α*D)
*Δ*A*Δ*C60 Ssa1p	382–582	SBD, CTD (*α*A-C)
*Δ*A*Δ*C88 Ssa1p	382–554	SBD, CTD (*α*A-B)
CTD Ssa1p	523–642	CTD
CTD*Δ*C20 Ssa1p	523–622	CTD (*α*B-D, part of rc)

**Figure 4.**
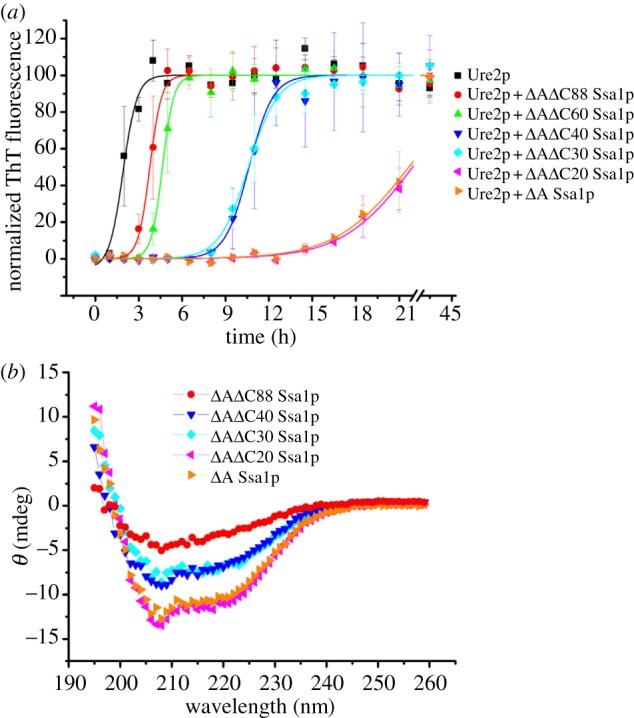
Effect of C-terminal deletion of *Δ*A Ssa1p on its inhibition of Ure2p amyloid fibril formation. (*a*) Fibril formation of 50 μM Ure2p in the absence or presence of 50 μM WT Ssa1p, *Δ*A Ssa1p, *Δ*A*Δ*C20 Ssa1p, *Δ*A*Δ*C30 Ssa1p, *Δ*A*Δ*C40 Ssa1p, *Δ*A*Δ*C60 Ssa1p or *Δ*A*Δ*C88 Ssa1p, as indicated. Incubation was in a Sim International SI-300 incubator at 30°C in phosphate buffer pH 8.4. Fibril formation was monitored by ThT fluorescence assay. The error bars represent the standard error of the mean. A sigmoidal fit of the data is shown. (*b*) Far-UV circular dichroism spectra of Ssa1p mutants: 4 μM *Δ*A Ssa1p, *Δ*A*Δ*C20 Ssa1p, *Δ*A*Δ*C30 Ssa1p, *Δ*A*Δ*C40 Ssa1p and *Δ*A*Δ*C88 Ssa1p, as indicated.

Having established that the N-terminal ATPase domain is not essential for inhibition, we truncated the C-terminal region of Ssa1p, in order to further define the role of different parts of the Ssa1p structure on inhibition of fibril formation. Five double truncation mutants were constructed: *Δ*1–381*Δ*623–642 Ssa1p, *Δ*1–381*Δ*613–642 Ssa1p, *Δ*1–381*Δ*603–642 Ssa1p, *Δ*1–381*Δ*583–642 Ssa1p and *Δ*1–381*Δ*555–642 Ssa1p, which, in addition to lacking the N-terminal ATPase domain, also lack 20, 30, 40, 60 or 88 amino acids, respectively, from the C-terminus. For convenience, we therefore refer to these mutants as *Δ*A*Δ*C20 Ssa1p, *Δ*A*Δ*C30 Ssa1p, *Δ*A*Δ*C40 Ssa1p, *Δ*A*Δ*C60 Ssa1p and *Δ*A*Δ*C88 Ssa1p, respectively (table 1). We then performed Ure2p fibril formation experiments in the presence of each of these mutants (figure 4*a*). The effect of *Δ*A*Δ*C20 Ssa1p was found to be similar to *Δ*A Ssa1p, whereas the effect of further truncation of the C-terminal region reduced the inhibition effect on Ure2p fibril formation in the following pattern: WT Ssa1p ≈ *Δ*A Ssa1p ≈ *Δ*A*Δ*C20 Ssa1p > *Δ*A*Δ*C30 Ssa1p ≈ *Δ*A*Δ*C40 Ssa1p > *Δ*A*Δ*C60 Ssa1p > *Δ*A*Δ*C88 Ssa1p. This suggests that truncation of the C-terminal 20 residues of Ssa1p has few structural or functional consequences with regard to this assay, whereas truncation of 30, 40, 60 or 88 residues causes more significant perturbation of structure and/or removal of residues that contribute to effective inhibition of fibril formation. This is consistent with the predicted structure of Ssa1p, in which the C-terminal truncation site for *Δ*A*Δ*C20 Ssa1p is located in the random coil, whereas the truncation sites for *Δ*A*Δ*C40 Ssa1p, *Δ*A*Δ*C60 Ssa1p and *Δ*A*Δ*C88 Ssa1p are located within the α-helix bundle, and *Δ*A*Δ*C30 Ssa1p is truncated close to the border between the random coil and helix bundle (figure 1). *Δ*A*Δ*C88 Ssa1p, which has the weakest effect, lacks helices C and D of the α-helix bundle, whereas *Δ*A*Δ*C60 Ssa1p and *Δ*A*Δ*C40 Ssa1p contain helix C, but all or part of helix D is truncated (figure 1), indicating that α-helices C and D each contribute individually to the ability of the SBD of Ssa1p to inhibit fibril formation of Ure2p. Interestingly, *Δ*A*Δ*C30 Ssa1p shows a similar degree of inhibition as *Δ*A*Δ*C40 Ssa1p, although from the predicted structure, *Δ*A*Δ*C30 Ssa1p is expected to contain an intact helix D. The far-UV CD spectra of *Δ*A*Δ*C30 and *Δ*A*Δ*C40 Ssa1p were observed to be similar to each other, but substantially different to that of *Δ*A and *Δ*A*Δ*C20 Ssa1p (figure 4*b*). This suggests that the residues in the border region up to and including residue 612 (the truncation point for *Δ*A*Δ*C30 Ssa1p) contribute to the stability and/or structural integrity of helix D of the α-helix bundle.

**Figure 5.**
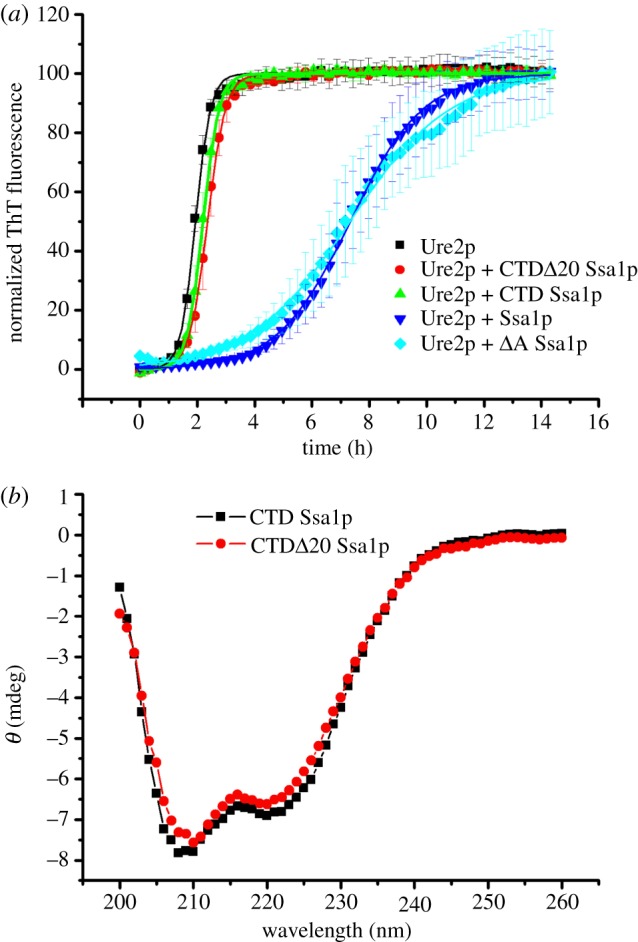
Effect of the isolated Ssa1p C-terminal α-helical bundle on inhibition of Ure2p amyloid fibril formation. (*a*) Fibril formation of 30 μM Ure2p in the absence or presence of 30 μM CTD Ssa1p or CTD*Δ*C20 Ssa1p, as indicated. Data in the presence of 30 μM Ssa1p and *Δ*A Ssa1p are also shown for comparison. Incubation was in a Perkin-Elmer EnSpire multimode plate reader at 30°C in phosphate buffer pH 8.4. Fibril formation was monitored by *in situ* measurement of ThT fluorescence. The error bars represent the s.e. of the mean. A sigmoidal fit of the data is shown. (*b*) Far-UV circular dichroism spectra of Ssa1p mutants: 10 μM CTD Ssa1p and CTD*Δ*C20 Ssa1p, as indicated.

In order to test whether the α-helix bundle within the CTD of Ssa1p is able to bind and inhibit fibril formation of Ure2p independently of the SBD, we constructed the mutant CTD Ssa1p; and also the mutant CTD*Δ*C20 Ssa1p, which contains the intact α-helical bundle, but lacks the final 20 residues of random coil (figure 1 and table 1). As shown in figure 5*a*, the CTD mutants of Ssa1p had negligible effect on the fibril formation of Ure2p, although the CD spectrum of these CTD mutants confirms they are folded and helical (figure 5*b*). While this does not rule out a direct interaction between the Ssa1p α-helix bundle and Ure2p, it does indicate that the influence of the C-terminal helical region on binding of the Ure2p substrate is dependent on the presence of the SBD.

Overall, our results show that the NBD and the C-terminal random coil regions of Ssa1p do not contribute to inhibition of Ure2p fibril formation, whereas further truncation of the C-terminal region reduces the inhibition ability of Ssa1p, with helices C and D of the α-helix bundle each contributing to the inhibition effect. On the other hand, the ability of the α-helix bundle to influence inhibition of Ure2p fibril formation is dependent on the presence of the SBD. This suggests that the structural basis of the ability of Ssa1p to inhibit Ure2p fibril formation resides in the SBD of Ssa1p, but that the structural integrity of helices C and D in the C-terminal region also contributes.

### (c) An inhibition model based on interaction between Ssa1p and native Ure2p and/or Ure2p fibril seeds can fit the kinetic course of Ure2p fibril formation in the presence of Ssa1p or *Δ*A Ssa1p

Chaperone binding to the soluble protein or to fibrillar species can affect any of the multiple steps in the assembly pathway, which include the formation of new aggregates from soluble protein (primary nucleation), the elongation of aggregates and the fragmentation of aggregates. The time course of fibril formation is largely determined by the rates of elongation and fragmentation of fibrils, and not by the rate of primary nucleation events, which enters only as logarithmic corrections in the integrated rate laws [70,83]. Owing to the predominant dependence of the reaction time course on the elongation and fragmentation rates, any change in the amount of available fibrillar ends and/or soluble protein owing to chaperone binding should be reflected primarily in a reduced elongation rate. Although interaction of the chaperone with the soluble protein may also affect the rate of primary nucleation, the corresponding effect on the experimental reaction time courses will be much less significant in comparison with the effect of comparable changes in the elongation rate.

In order to test this idea, we performed a global fit of the fibril formation data measured in the presence of varying concentrations of Ssa1p (figure 2*a*) with an elongation rate constant that was allowed to vary for each experiment but where the primary nucleation rate constant and the fragmentation rate constant were held fixed for the entire dataset. With this constraint, the data were fitted to an analytical solution [70–72] of the kinetics of breakable filament assembly (see §2), which describes fibril formation in terms of three rate constants: nucleation (*k*_n_), fibril elongation (*k*_+_) and fibril fragmentation (*k*_−_). This approach accounts well for the observed trends as can be seen in figure 2*a*. The inset in figure 2*a* shows the variation with Ssa1p concentration of the elongation rate obtained from the fits of the data. A similar result was obtained by fitting the data obtained in the presence of *Δ*A Ssa1p (figure 2*b*, inset). This result indicates that inhibition of Ure2p amyloid fibril formation by Ssa1p or *Δ*A Ssa1p is due primarily to an effect on the rate of fibril elongation.

### (d) Observation of an interaction between *Δ*A Ssa1p and Ure2p during the course of fibril formation by biosensor measurements

**Figure 6.**
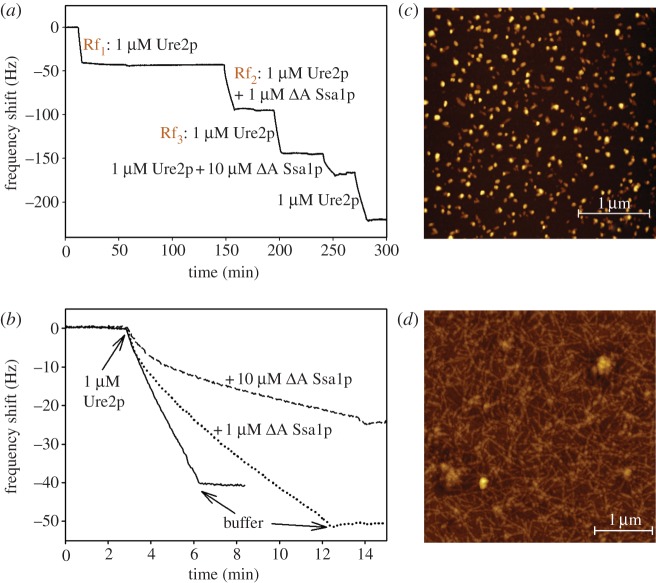
QCM measurements of Ure2p fibril growth in the presence and absence of *Δ*A Ssa1p. The rate of decrease of resonant frequency of the quartz crystal is proportional to the elongation rate of the surface-bound seed fibrils. (*a*) This sequence of QCM measurements demonstrates that the elongation rate in the presence of a mixture of soluble Ure2p and *Δ*A Ssa1p is slower (Rf_2_) than in the absence of the chaperone (Rf_1_). Renewed incubation with pure Ure2p (Rf_3_) leads to an increased rate compared with that during Rf_2_. However, the rate does not recover fully to the one observed during Rf_1_, indicating that in addition to the inhibitory effect of an interaction between the chaperone and the soluble Ure2p, the chaperone is also likely to interact with the seed fibrils on the sensor. (*b*) Direct comparison between the elongation rates of the seeds with 1 μM soluble Ure2p in the presence of 0, 1 or 10 μM *Δ*A Ssa1p. (*c*) AFM image of Ure2p seeds before commencement of the QCM fibril growth experiment. (*d*) AFM image of Ure2p fibrils on the QCM sensor after an experiment, such as the one shown in (*a*).

To obtain independent confirmation of the results from the ThT kinetic assays, we carried out surface-bound growth experiments with a quartz crystal microbalance. The principle of these experiments is to monitor the elongation of surface-bound amyloid fibrils via changes in the resonant frequency of the quartz crystal sensor, which are proportional to the mass increase of the surface-bound fibrils [76]. If the fibrils are irreversibly attached to the QCM-sensor surface, then the growth of a constant ensemble can be monitored under varying conditions [73,75], for example, the presence and absence of a molecular chaperone. When surface-bound Ure2p amyloid fibril seeds are brought into contact with a solution of Ure2p, the elongation of these seeds leads to a decrease in resonant frequency (figure 6*a*). The rates of change of frequency are proportional to the elongation rates and can be directly compared for different conditions (figure 6*b*). AFM imaging of the QCM sensor before and after an experiment confirms that the observed increase in mass corresponded to lengthening of the fibrils (figure 6*c,d*).

The reproducibility of this assay of amyloid elongation kinetics was confirmed by repeated incubation of the QCM sensor with a solution of Ure2p under identical conditions (see the electronic supplementary material, figure S3*a*). The maximum variability of the measured growth rates was found to be 30 per cent and so we concluded that changes in rate owing to a change in solution conditions larger than 30 per cent are significant. Before being able to test the influence of a chaperone on the growth rates of the surface-bound fibrils, we also needed to minimize the binding of the chaperone to the surface of the quartz crystal, because the induced frequency shifts stemming from fibril growth and chaperone binding are difficult to disentangle. To test the extent of chaperone binding in the absence of Ure2p fibril seeds, we applied WT Ssa1p or *Δ*A Ssa1p onto empty sensors. WT Ssa1p showed significant binding to the empty sensor, whereas *Δ*A Ssa1p showed minimal non-specific binding (see the electronic supplementary material, figure S3*b*). Therefore, only *Δ*A Ssa1p was used in further biosensor experiments.

When the growth rates of the Ure2p amyloid fibrils with and without stoichiometric amounts of *Δ*A Ssa1p were compared, it was found that the presence of the chaperone decreased the fibril growth rate by a factor of 2.3 (figure 6 and table 2), which is significant according to the reproducibility experiments described earlier. Furthermore, subsequent incubation of the fibrils with pure Ure2p solution led to a growth rate lower than that observed before treatment with the chaperone, with the ratio of rates before and after chaperone treatment being 1.5 (table 2). The observation that the initial rate was not quantitatively recovered can be explained by an interaction between the chaperone and the seed fibrils on the QCM sensor, leading to inhibition even in the absence of soluble chaperone. A similar but stronger inhibitory effect was observed when the soluble Ure2p protein was mixed with a 10-fold excess of *Δ*A Ssa1p; here, the elongation rate was reduced by a factor of 4.5 compared with the rate in the absence of chaperone (figure 6 and table 2). Thus, our label-free QCM experiments independently confirmed that *Δ*A Ssa1p inhibits the growth of Ure2p fibrils. The mechanism is presumably twofold, involving interaction between *Δ*A Ssa1p with both soluble and fibrillar Ure2p.

**Table 2.** Inhibition effect of *Δ*A Ssa1p on Ure2p fibril growth monitored by QCM.

factor^a^	*Δ*A Ssa1p (1:1 with Ure2p)	*Δ*A Ssa1p (10:1 with Ure2p)	lysozyme^b^	bovine beta lactoglobulin^b^
Rf_1_/Rf_2_	2.3 ± 0.2	4.5 ± 0.5	1.2	1.1
Rf_1_/Rf_3_	1.5 ± 0.2	1.4 ± 0.3	n.a.	1.02

^a^The original rate of the oscillation frequency change when applying only Ure2p solution was defined as Rf_1_, the rate of oscillation frequency change when applying the mixture of Ssa1p domain-deletion mutants and Ure2p was defined as Rf_2_, and the rate of oscillation frequency change when applying Ure2p alone again is Rf_3_ (figure 6). The value of Rf_1_/Rf_2_, which represents the extent of inhibition due to the presence of chaperone, is displayed. The value of Rf_1_/Rf_3_, which represents the extent to which the fibrils and/or sensor has been modified by previous application of chaperone, is also displayed.

^b^Control experiments were performed with lysozyme or bovine beta lactoglobulin in place of chaperone. The molecular weights of *Δ*A Ssa1p, lysozyme and bovine beta lactoglobulin are 31, 14 and 18 kDa, respectively.

To confirm that the observed inhibition was due to a specific interaction between Ure2p and Ssa1p, we performed control experiments in which lysozyme and bovine beta lactoglobulin replaced the chaperone. No significant inhibition of fibril elongation could be detected in either case (table 2).

### (e) Observation of an interaction between *Δ*A Ssa1p and native Ure2p in solution by dynamic light scattering

**Figure 7.**
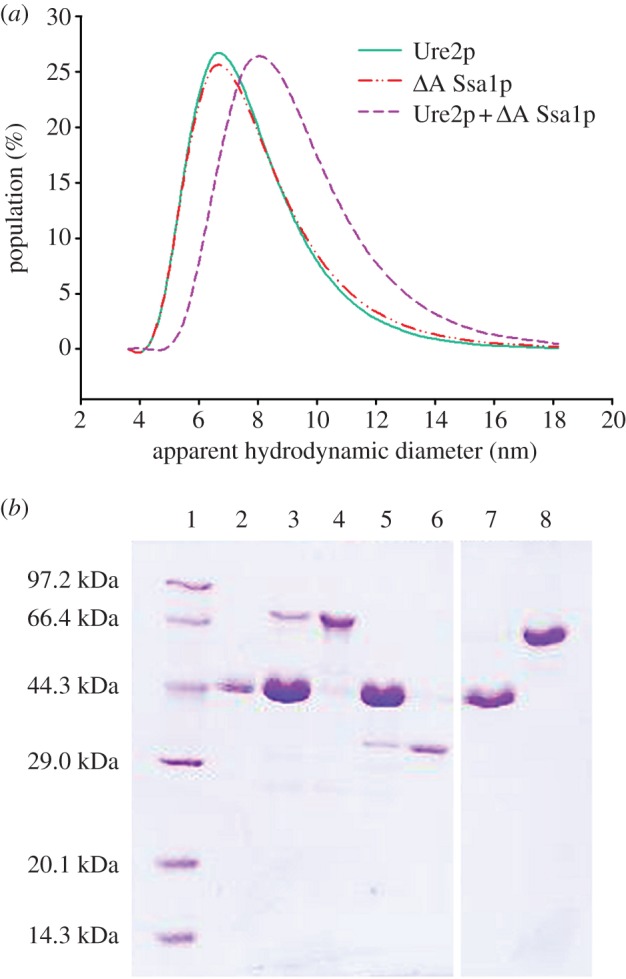
Interaction between Ure2p and Ssa1p. (*a*) Interaction between native Ure2p and *Δ*A Ssa1p observed by dynamic light scattering measurements. Solid line: Ure2p; dashed-dotted line: *Δ*A Ssa1p; dashed line: a 1 : 1 mixture of Ure2p and *Δ*A Ssa1p. (*b*) Interaction of Ure2p seeds with WT Ssa1p or *Δ*A Ssa1p observed by the filament pull-down assay as described in the text. The pellets formed after high-speed centrifugation in 40% sucrose were resuspended in buffer and loaded onto a 12% SDS–PAGE gel. Lane 1, low molecular mass marker; lanes 2, 3, 5 and 7: Ure2p seeds incubated alone or with WT Ssa1p, *Δ*A Ssa1p or BSA, respectively; lanes 4, 6 and 8: WT Ssa1p, *Δ*A Ssa1p or BSA, respectively, as the molecular weight control.

In order to investigate further whether *Δ*A Ssa1p interacts directly with native Ure2p in solution, we used DLS measurements. The diffusion of particles in solution induces fluctuations in the intensity of scattered light. These fluctuations, which are sensitive to particle size, can be detected by DLS and provide an indication of the distribution of different-sized molecules within a population. When solutions containing Ure2p, *Δ*A Ssa1p or a mixture of both proteins were analysed by DLS, differences in the hydrodynamic diameters of proteins in the mixture compared with those in pure preparations were observed. This suggests that Ure2p and *Δ*A Ssa1p interact directly in solution to form a complex (figure 7*a*).

### (f) Observation of an interaction between Ssa1p and Ure2p fibril seeds by pull-down assay

In order to investigate further whether Ssa1p and *Δ*A Ssa1p interact directly with Ure2p fibril seeds, we used a pull-down assay. Ure2p fibrils were fragmented by sonication to form short fibril seeds, which were incubated with WT Ssa1p or *Δ*A Ssa1p. Fibrils were harvested by high-speed centrifugation and resuspended for SDS–PAGE analysis. In general, oligomeric seeds of Ure2p are found in the pellet fraction, whereas soluble WT Ssa1p or *Δ*A Ssa1p appear in the supernatant fraction (data not shown). However, when the Ure2p seeds were incubated with WT Ssa1p or *Δ*A Ssa1p, bands corresponding to Ssa1p were observed in the pellet fraction (figure 7*b*, lanes 3 and 5). This indicates that WT Ssa1p and *Δ*A Ssa1p interact directly with Ure2p fibril seeds.

## 4. Discussion

A number of recent studies indicate that molecular chaperones exert important influence on amyloid fibril formation and prion propagation [52,59,79,80,84,85]. Overexpression of the HSP70 homologue Ssa1p is able to cure the [*URE3*] prion state in yeast cells [50,86,87] and Ssa1p can inhibit fibril formation in the presence of ATP *in vitro* [57,58]. Here, we demonstrate that Ssa1p inhibits fibril formation of Ure2p in the presence or absence of ATP or ADP. We show that inhibition is primarily due to the effect of Ssa1p on the elongation rate of Ure2p fibril growth, rather than by an effect on nucleation or breakage of fibrils. We observe interaction between Ssa1p and native Ure2p as well as Ure2p fibril seeds. Previous results also suggest interaction between Ssa1p and species that are formed as intermediates in the course of Ure2p fibril formation [57].

HSP70 is involved in a number of important functions and is essential for cell viability, so cannot be deleted or substantially truncated *in vivo* [23], and the structural basis of functional roles is even harder to ascertain. In this study, we used the power of *in vitro* experiments to dissect out the structural basis for the role of HSP70 in the regulation of prion propagation, focusing on the protein determinant of the [*URE3*] prion state, Ure2p. It has been reported that mutations in the NBD of Ssa1p affect [*URE3*] propagation *in vivo*, but not the process of [*URE3*] elimination [58], suggesting that these processes have different requirements for HSP70 activity. This is consistent with our *in vitro* experiments, which mimic the process of prion elimination. However, although we do not observe any significant difference in inhibition of Ure2p fibrillation by Ssa1p with or without ATP/ADP *in vitro*, we do not discount the possibility that this process may indeed be nucleotide-dependent *in vivo*, where a range of co-chaperones and cofactors (such as HSP40 and HSP104) cooperate with and regulate HSP70 function.

As chaperones operate as a cooperative network, then the role and mechanism of individual chaperones is difficult to dissect *in vivo*. Because HSP70 cooperates with the co-chaperone HSP40, we also checked the combined effect of Ssa1p and the yeast HSP40 family member, Ydj1p. Ydj1p is reported to cure [*URE3*] by overexpression *in vivo* [88] and to inhibit Ure2p fibril formation *in vitro* [57,59]. However, the fact that mutation of the J domain of Ydj1p impairs its ability to cure [*URE3*] led to the suggestion that the curing effect of Ydj1p occurs indirectly via its stimulation of HSP70 [79,80]. The Ydj1p mutant H34Q is deficient in both stimulation of the chaperone activity of Ssa1p [81,82] and curing of [*URE3*] [79,80]. Our data show that Ydj1p H34Q shows significantly weaker inhibition of Ure2p fibrillation compared with WT Ydj1p *in vitro*, which accounts for the reduced extent of curing in the presence of Ssa1p (figure 3) as well as impaired curing *in vivo* [79,80]. Therefore, the role of a direct interaction between Ydj1p and Ure2p in [*URE3*] curing clearly cannot be excluded on the basis of the *in vivo* data. Ydj1p may interact with Ure2p and play a direct role in curing of [*URE3*], in addition to its role in activating the ATPase activity of Ssa1p.

The C-terminal lid of the HSP70 SBD fluctuates between open and closed conformations. This structural transition is regulated allosterically by ATP binding and hydrolysis in the NBD and modulates the affinity of HSP70 for substrate. The nucleotide-free and ADP states are observed to be relatively heterogeneous, whereas ATP (or ATP-analogue) bound states show less variability in conformation and the open state is preferred [30,89–92]. However, open and closed conformations are populated in all nucleotide states (ATP, ADP and nucleotide-free), albeit in different proportions [45,92,93]. Further, substrates are involved in determining the conformation, with protein substrates favouring a more open conformation than small peptide substrates [45,91]. It has been proposed that the differences in affinity observed in different nucleotide states are not in fact due to different conformations *per se*, but due to the frequency of fluctuation between the open and closed states and their relative abundance [93]. Much of our understanding of the HSP70 ATPase cycle and its associated dynamic conformational changes has come from studies using small peptide substrates, with *E. coli* DnaK being the most intensively studied HSP70 homologue to date. Important differences in the regulatory mechanism have been observed even between closely related HSP70 homologues [91,92], meaning that extrapolation between different systems must be done with caution. The lack of structural data for the yeast homologue Ssa1p is a significant limitation. Further, there is clear evidence from genetic studies for differences in the way that ‘normal’ (e.g. heat denatured) and amyloidogenic or prion substrates are recognized [51]. Nevertheless, in the light of current understanding of HSP70 structure and function, the lack of nucleotide (or even NBD) dependence observed here suggests that the conformational changes driven by nucleotide binding and hydrolysis are unimportant on the timescale of the Ure2p fibril formation experiments and/or that binding to Ure2p locks HSP70 into the preferred conformation for binding, overriding the conformational preference of any given nucleotide state.

Having found that the presence or absence of nucleotide had no significant effect on the ability of Ssa1p to inhibit Ure2p fibril formation, we tested whether or not entirely deleting the ATPase domain reduced the inhibition ability of Ssa1p towards Ure2p and found it did not. We found that the ATPase domain-deletion mutant (*Δ*A Ssa1p) interacted with Ure2p in a similar manner to the full-length protein. We then went on to examine the effect of progressive truncation of the Ssa1p C-terminal domain, in order to further refine the structural regions that are important for the inhibition effect on Ure2p.

The C-terminal lid is thought to contribute to the oligomerization of Ssa1p [94] and provide an acidic environment for the HSP70 client. When the client protein enters the groove formed by the β-sheet of the SBD, the C-terminal lid closes the exit [43,44]. The first α-helix in the α-helix bundle, α-helix B, swings upwards to form a continuous helix with α-helix A (the first α-helix in the CTD) resulting in complete exposure of the substrate binding groove [95]. Recently, Mayer and co-workers [45] proposed that the lid contacts directly with the substrate and can undergo different conformational changes depending on the size of substrate. In this study, when the C-terminal final 20 residues comprising part of the random coil part of the lid domain were deleted, there was no effect on the ability of Ssa1p to inhibit Ure2p fibril formation *in vitro*. Further deletions around the border between the C-terminal random coil region and the α-helical bundle reduced the ability of Ssa1p to inhibit fibril formation, indicating that this region may contribute to the structural integrity of the α-helical bundle. Successive deletion of helices D and C of the α-helical bundle resulted in incremental weakening of the inhibition effect of Ssa1p on Ure2p fibril formation. On the other hand, the α-helical bundle of Ssa1p did not influence fibril formation of Ure2p in isolation from the SBD. Taken together, this indicates that the SBD of Ssa1p is primarily responsible for inhibition of Ure2p fibril formation, and the α-helical bundle of the CTD facilitates this effect. Interestingly, our results suggest that the α-helical bundle does not have to be fully formed to contribute to this effect. These findings are similar to the inhibitory effect of human HSP70 on aggregation of α-synuclein, which also relies primarily on the action of the SBD, but is facilitated by the presence of the CTD [47].

Ssa1p inhibition of fibril formation by Ure2p is efficient when Ssa1p is added during the lag phase or early exponential phase of fibril growth, suggesting that Ssa1p interacts with species present during these phases of growth. Our DLS data indicate that *Δ*A Ssa1p interacts with the native Ure2p protein in solution which is consistent with previous work that proposed an interaction between Ssa1p and the native Ure2p protein [57,60]. We also used a pull-down assay [47,96,97] to detect the interaction between fibrils and Ssa1p. WT Ssa1p and *Δ*A Ssa1p co-pelleted with Ure2p filament seeds, which provides an explanation for the delay in the lag time observed in the ThT experiments. Results from the pull-down assay also help to explain the incomplete recovery of the elongation rate after chaperone treatment observed by QCM, indicating that *Δ*A Ssa1p interacts with Ure2p fibril seeds, forming complexes that could not be completely dissociated by washing. The small amount of chaperone that remained bound to seeds on the QCM sensor reduced the growth rate of Ure2p fibrils. HSP70 has been proposed to adopt an ‘intermediate-open’ conformation upon binding to bulky substrates such as protofibrils or fibrils, where the substrate binding groove is not completely sealed by the lid [45]. In this case, Ssa1p presumably adopts an ‘intermediate-open’ conformation to interact with Ure2p fibril seeds.

Taken together, our results indicate that WT and *Δ*A Ssa1p are able to interact directly with both native Ure2p and Ure2p fibril seeds, providing an explanation for the observation of inhibition of fibril formation even in the absence of nucleotide. We show that this inhibitory effect is primarily reflected in a reduction in the rate of fibril elongation. Further, our results concerning the involvement of the HSP70 CTD in binding are consistent with other recent results that indicate that the SBD is crucial for substrate interactions, but binding is enhanced by the presence of the CTD, either due to direct interaction between the substrate and the CTD, or due to the action of the CTD as a lid to facilitate capture of substrate molecules.
